# External dsRNA Downregulates Anthocyanin Biosynthesis-Related Genes and Affects Anthocyanin Accumulation in *Arabidopsis thaliana*

**DOI:** 10.3390/ijms22136749

**Published:** 2021-06-23

**Authors:** Konstantin V. Kiselev, Andrey R. Suprun, Olga A. Aleynova, Zlata V. Ogneva, Alexander V. Kalachev, Alexandra S. Dubrovina

**Affiliations:** 1 Laboratory of Biotechnology, Federal Scientific Center of the East Asia Terrestrial Biodiversity, Far Eastern Branch of the Russian Academy of Sciences, 690022 Vladivostok, Russia; kiselev@biosoil.ru (K.V.K.); SUPRUN@biosoil.ru (A.R.S.); aleynova@biosoil.ru (O.A.A.); ogneva@biosoil.ru (Z.V.O.); 2 Laboratory of Embryology, A.V. Zhirmunsky National Scientific Center of Marine Biology, Far Eastern Branch of the Russian Academy of Sciences, 690041 Vladivostok, Russia; akalachev@imb.dvo.ru

**Keywords:** exogenous dsRNA, plant foliar treatment, plant gene regulation, RNA interference, gene silencing, anthocyanins

## Abstract

Exogenous application of double-stranded RNAs (dsRNAs) and small-interfering RNAs (siRNAs) to plant surfaces has emerged as a promising method for regulation of essential genes in plant pathogens and for plant disease protection. Yet, regulation of plant endogenous genes via external RNA treatments has not been sufficiently investigated. In this study, we targeted the genes of chalcone synthase (CHS), the key enzyme in the flavonoid/anthocyanin biosynthesis pathway, and two transcriptional factors, MYBL2 and ANAC032, negatively regulating anthocyanin biosynthesis in *Arabidopsis*. Direct foliar application of *AtCHS*-specific dsRNAs and siRNAs resulted in an efficient downregulation of the *AtCHS* gene and suppressed anthocyanin accumulation in *A. thaliana* under anthocyanin biosynthesis-modulating conditions. Targeting the *AtMYBL2* and *AtANAC032* genes by foliar dsRNA treatments markedly reduced their mRNA levels and led to a pronounced upregulation of the *AtCHS* gene. The content of anthocyanins was increased after treatment with *AtMYBL2-*dsRNA. Laser scanning microscopy showed a passage of Cy3-labeled *AtCHS*-dsRNA into the *A. thaliana* leaf vessels, leaf parenchyma cells, and stomata, indicating the dsRNA uptake and spreading into leaf tissues and plant individual cells. Together, these data show that exogenous dsRNAs were capable of downregulating *Arabidopsis* genes and induced relevant biochemical changes, which may have applications in plant biotechnology and gene functional studies.

## 1. Introduction

The current RNA-based crop improvement studies employ the RNA interference (RNAi) phenomenon for downregulation of gene targets in plants for further plant disease control and crop management [1,2]. The principles of the RNAi phenomenon are also being actively exploited in plant gene functional studies. RNAi is a natural regulatory mechanism that involves sequence-specific degradation of target mRNAs or translation inhibition induced by short small-interfering RNAs (siRNAs) or microRNAs (miRNAs) originated from long double-stranded RNA (dsRNA) precursors that may vary in length and origin [3,4]. Plants have developed RNAi as an effective mechanism implicated in plant pathogen and viral defense [5], plant growth and development [6], and abiotic stress responses [7]. In the course of RNAi, long dsRNAs precursors are recognized and processed by a ribonuclease DICER into small RNA duplexes of 20–24-nucleotide (nt)-long, i.e., siRNAs and miRNAs [3]. These siRNAs are then incorporated into the RNA-induced silencing complex (RISC) that drives silencing of the target mRNAs via their cleavage, destabilization, or hindering translation.

The major RNAi-based crop improvement/protection strategies include the generation of dsRNA/hairpin RNA (hpRNA)-expressing transgenic plants and host-induced gene silencing (HIGS), which allowed the silencing of genes in plant microbial pathogens [8,9,10] or application of modified plant viruses inducing degradation of target plant mRNA, i.e., virus-induced gene silencing or VIGS [11]. RNAi-based insect management strategies include generation of transgenic plants expressing dsRNAs targeting essential insect genes, feeding insects with dsRNAs, or plant foliar treatments with dsRNAs [12,13]. However, the consequences of plant genetic modifications are not clear, which raises serious public concerns on their impacts on human health and environment [14]. Furthermore, generation of transgenic plants is a costly and a complicated process for many horticultural crops. Although VIGS does not require genetic modifications of plants, this method possesses serious limitations that prevent its wide application [15]. Therefore, development of an alternative approach for plant gene regulation without genomic modifications is an important challenge for plant biotechnology.

There is an increasing number of studies that show induction of plant fungal [16,17,18,19,20,21] and viral [22,23,24,25,26,27] resistance after external application (spraying or mechanical inoculation) of dsRNAs, siRNAs, or hpRNAs designed to target virulence-related genes of the pathogens. Recent studies have also provided evidence that both plants and infecting pathogens were capable of the RNA uptake, and this eventually triggered RNAi-mediated silencing of the pathogen virulence-related genes [16,17,21,22,27]. Exogenously induced RNAi has recently emerged as a strategy with a potential to protect plants from microbial diseases, viral infections, and invading insects [9,28,29].

Several studies reported that external application of dsRNAs [22,30,31] to *Arabidopsis thaliana* and siRNAs [32,33,34] to *A. thaliana* or *Nicotiana benthamiana* triggered silencing of common plant transgenes, such as green fluorescent protein (*GFP*), β-glucuronidase (GUS), yellow fluorescent protein (YFP), or neomycin phosphotransferase II (*NPTII*). Plant transgenes are known to be more prone for RNAi-mediated suppression in comparison with plant endogenes [35,36,37], and, therefore, targeting transgenes might be more achievable. However, there are also data showing that naked *GFP*-dsRNAs and *GFP*-hpRNAs did not induce *GFP* silencing in *N. benthamiana* and were not processed into siRNAs, indicating insufficient dsRNA uptake by plant cells [38,39]. According to Numata et al. [32] and Dalakouras et al. [33], downregulation of the *GFP* and *YFP* transgenes after foliar application of siRNAs was successful only after application of accessory technologies (using carrier peptide or high-pressure spraying). According to our recent data, appropriate plant age, late time of day, low soil moisture (at the moment of dsRNA application), and optimal dsRNA application modes were important for efficient *NPTII* transgene suppression in *A. thaliana* induced by direct foliar dsRNA treatments [31].

To the best of our knowledge, there are four investigations and a patent that showed that external plant treatments with naked dsRNAs led to downregulation of plant endogenous genes, including silencing of the 3-phosphate synthase (*EPSPS*) gene in tobacco and amaranth leaves [40], *MYB1* gene in the orchid flower buds [41], *Mob1A*, *WRKY23*, and *Actin* genes in *Arabidopsis* and rice [42], two sugar transporter genes *STP1* and *STP2* in tomato seedlings [43], and a downy mildew susceptibility gene *LBDIf7* in grapevine [44]. According to the data, external plant dsRNA treatments led to the dsRNA uptake, reduced mRNA levels of the gene targets, and some phenotypic or biochemical changes. There were also experimental findings where nanoparticles [45] and laser light [46] were used to ensure perception of exogenous dsRNA and downregulation of the targeted endogenous genes *SHOOT MERISTEMLESS* (*STM*) and *WEREWOLF* (*WER*) in *A. thaliana* and phytoene desaturase (*PDS*) gene in *Citrus macrophylla* after external dsRNA treatments.

**Figure 1:**
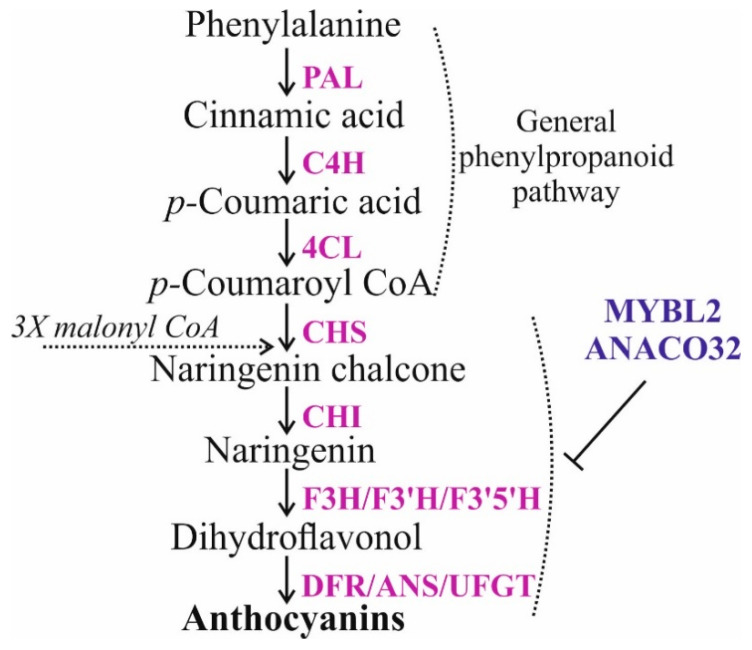
Schematic representation of the anthocyanin biosynthesis pathway. Enzymes of each step are shown in purple. Enzymes involved in general phenylpropanoid pathway are phenylalanine ammonia lyase (PAL), cinnamate 4-hydroxylase (C4H) and 4-coumaryol CoA ligase (4CL). Enzymes involved in flavonoid biosynthesis are chalcone synthase (CHS), chalcone isomerase (CHI), flavanone 3-hydroxylase (F3H), flavanone 3′5′-hydroxylase, (F3′5′H) and flavanone 3′-hydroxylase (F3′H). Anthocyanins are synthesized by dihydroflavonol 4-reductase (DFR), synthase (ANS), and UDP-glucose:flavonoid-3-O-glucosyltransferase (UFGT). Two transcriptional factors negatively regulating anthocyanin biosynthesis include a R3-type single-MYB protein (MYBL2) and a NAC-type transcription factor (ANAC032).

Anthocyanins are naturally occurring colored pigments derived from the plant phenylpropanoid pathway and are relatively easy to induce for accumulation and quantitative analysis [47,48]. Apart from providing color to plants and animal attraction, anthocyanins are known to possess beneficial human health effects and plant protective properties against pests and pathogens [47,49]. Therefore, in the present study, we targeted anthocyanin biosynthesis-related genes (Figure 1), including chalcone synthase (*CHS*) and two transcriptional repressors, a R3-type single-MYB protein *MYBL2* and a NAC-type transcription factor *ANAC032* genes, in *A. thaliana* by direct foliar treatments with dsRNAs and siRNAs. This analysis quantitatively documented changes in *AtCHS*, *AtMYBL2*, and *AtANAC032* gene expression and anthocyanin production after the foliar dsRNA and siRNA applications. To prove the specificity of the downregulation effect, we also treated wild-type *A. thaliana* with dsRNAs and siRNAs specific for the bacterial neomycin phosphotransferase II (*NPTII*) and did not observe an effect.

## 2. Results

### 2.1. AtCHS-Specific Exogenous dsRNAs Downregulate AtCHS mRNA Levels and Anthocyanin Content in Arabidopsis

**Figure 2:**
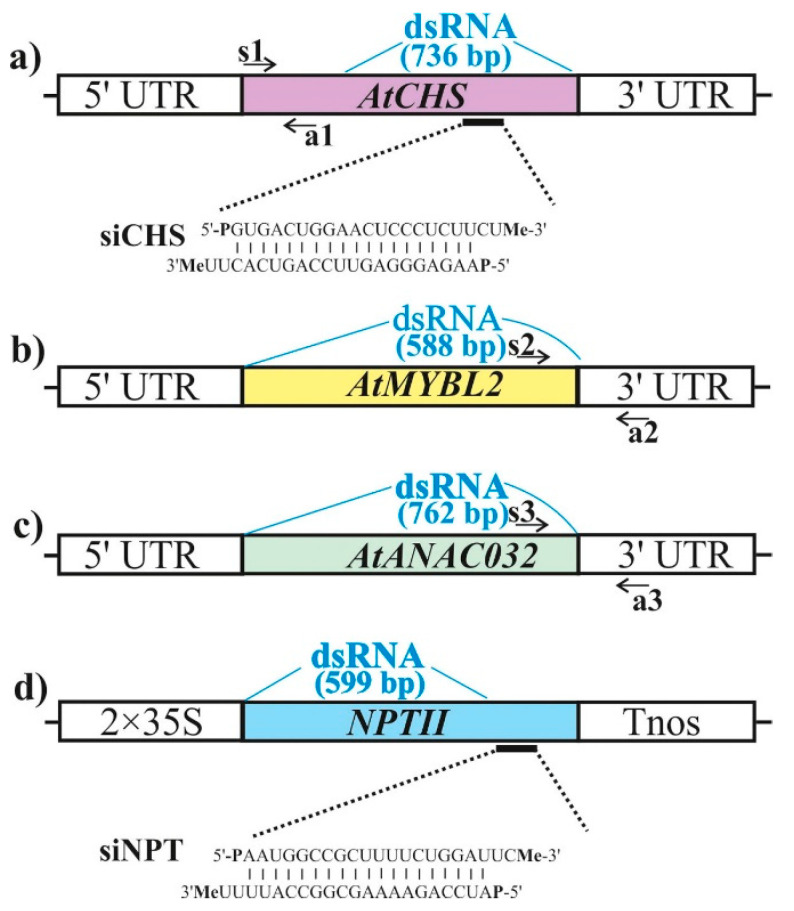
Schematic representation of the dsRNA position and siRNAs structures used in this study and the qRT-PCR primers designed to verify the effects of external RNA treatments on the levels of endogenous *AtCHS*, *AtMYBL2*, and *AtANAC032* mRNAs. (**a**) Representation of *AtCHS* cDNA coding region with positions of the *AtCHS-*specific dsRNA and siRNA; (**b**) representation of *AtMYBL2* cDNA coding region with position of the *AtMYBL2*-specific dsRNA; (**c**) representation of *AtANAC032* cDNA coding region and position of *AtANAC032*-specific dsRNA; (**d**) representation of *NPTII* coding region and positions of the *NPTII*-specific dsRNA and siRNA. Black arrows indicate positions of the primers (s1, a1, s2, a2, s3, a3) used for amplification of the endogenous *AtCHS*, *AtMYBL2*, and *AtANAC032* transcripts. UTR—untranslated region. 2 × 35S—the double 35S promoter of the cauliflower mosaic virus (CaMV). Tnos—nopaline synthase terminator.

We used PCR and in vitro transcription protocol to produce dsRNA molecules of the *AtCHS* gene, encoding the key enzyme in the flavonoid/anthocyanin biosynthesis pathway (Figure 1), and the non-related *NPTII* bacterial gene, encoding the bacterial neomycin phosphotransferase II enzyme. We synthesized the *NPTII*-dsRNAs and treated wild-type *A. thaliana* to verify whether any observed effects on *AtCHS* mRNA levels were sequence-specific. To analyze the effect of exogenous dsRNAs on the expression of *AtCHS*, large fragments of *AtCHS* and *NPTII* cDNAs were amplified (Figure 2a,d). Then, the obtained PCR products, containing T7 promoters at both ends, were used as templates for in vitro transcription. For external application, the synthesized dsRNAs were diluted in water to a final concentration of 0.35 µg/µL. The dsRNAs (100 µL of each dsRNA per individual plant, i.e., 35 µg) were applied on the leaf surface (on both the adaxial and abaxial sides) of four-week-old *A. thaliana* rosettes by spreading with sterile individual soft brushes [31] (Supplementary Video S1). Importantly, we treated the four-week-old rosettes of *A. thaliana* at a late day time (21:00–21:30) under low soil moisture conditions in all experiments, since the appropriate plant age, late day time, and low soil moisture at the time of dsRNA application were important parameters for successful *NPTII* suppression in transgenic *A. thaliana* according to our recent analysis [31]. An analysis of the dsRNA concentration effect on transgene silencing in *A. thaliana* was performed previously [30], indicating that 35 µg of a transgene-encoding dsRNA resulted in the highest transgene silencing efficiency as compared to other dsRNA concentrations.

**Figure 3:**
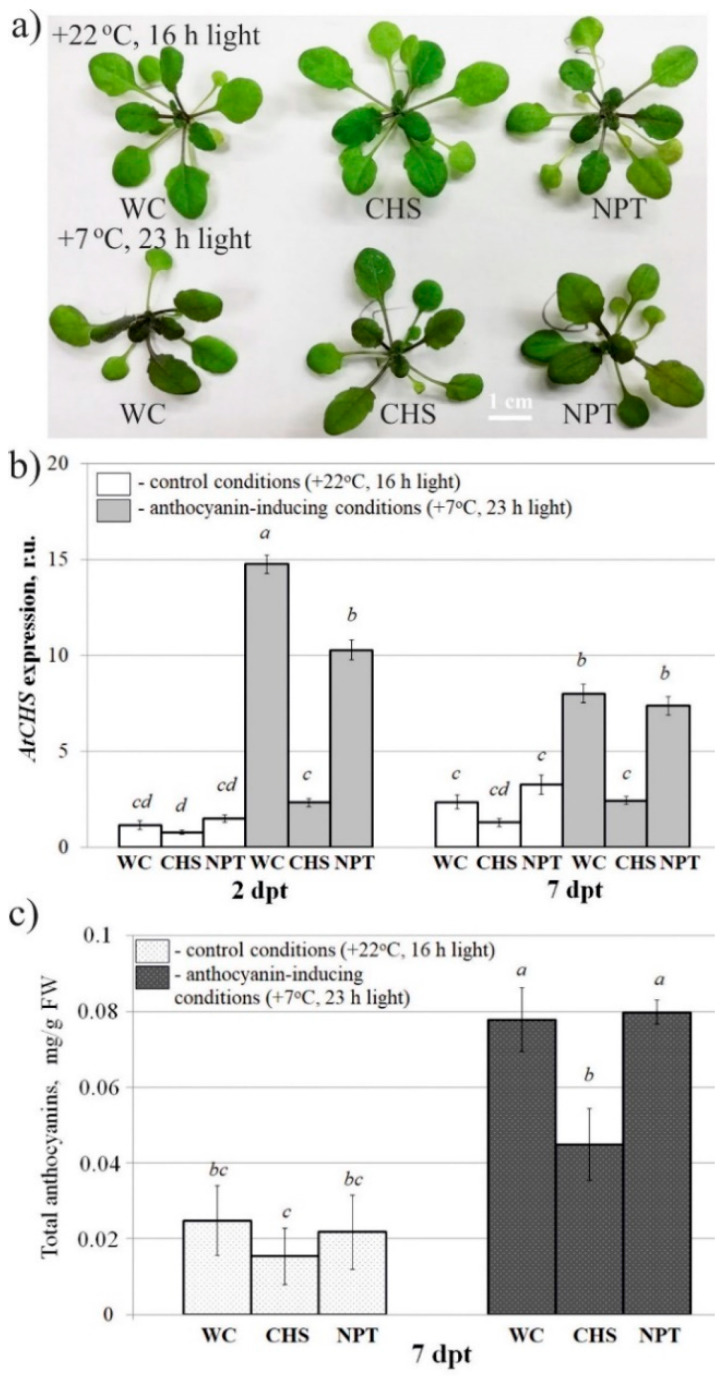
The effect of external *AtCHS*- and *NPTII*-encoding dsRNAs on *AtCHS* mRNA level and total anthocyanin content in *Arabidopsis thaliana*. (**a**) *Arabidopsis* plants grown under control (+22 °C, 16 h light, upper panel) and anthocyanin-inducing (+7 °C, 23 h light, lower panel) conditions for seven days after treatment with sterile water or synthetic dsRNA. (**b**) Quantitative real-time PCR measuring relative mRNA levels of endogenous *AtCHS* in the leaves of *A. thaliana* treated with water or synthetic dsRNAs. (**c**) HPLC results of total anthocyanins in the leaves of *A. thaliana* grown under the control and anthocyanin-inducing conditions. WC—*A. thaliana* treated with sterile water; CHS—*A. thaliana* treated with *AtCHS*-dsRNAs; NPT—*A. thaliana* treated with *NPTII*-dsRNA; dpt—days post-treatment. The data are presented as the mean ± SE (three independent experiments). Means followed by the same letter were not different using Student’s *t* test. *p* < 0.05 was considered statistically significant.

Then, we studied whether exogenous application of the naked *AtCHS* and *NPTII*-dsRNAs on the foliar surface of wild-type *A. thaliana* could led to any changes in the mRNA transcript levels of *AtCHS* gene and anthocyanin levels in comparison with the control water treatment (Figure 3). Since under standard cultivation conditions anthocyanin production and *AtCHS* expression were low, we divided the treated *A. thaliana* rosettes into two groups for post-treatment incubation either under control conditions (+22 °C, 16 h light) or anthocyanin-inducing conditions (+7 °C, and 23 h light) for two and seven days in order to induce *AtCHS* expression and anthocyanin biosynthesis (Figure 3a). qRT-PCR revealed that cultivation of *A. thaliana* under the anthocyanin-inducing conditions resulted in a dramatically higher *AtCHS* mRNA levels in the control plants treated with water and *NPTII*-dsRNAs than cultivation under control conditions (Figure 3b). Importantly, this *AtCHS*-induction effect was not observed for plants treated with *AtCHS*-dsRNAs under the anthocyanin-inducing conditions, at both two days and seven days post-treatment (Figure 3b). Notably, *AtCHS* mRNA levels were considerably lower in the *AtCHS*-dsRNA-treated *A. thaliana* than in the water- and *NPTII*-treated controls under the anthocyanin-inducing conditions, at both two days and seven days post-treatment.

**Figure 4:**
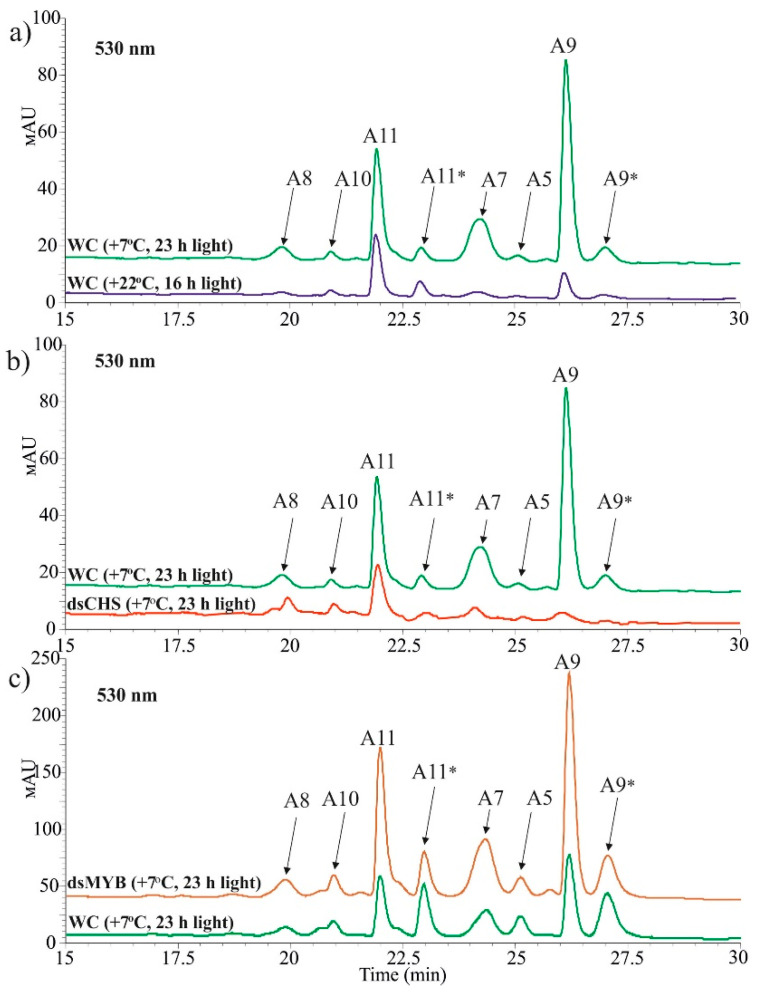
HPLC chromatograms of anthocyanins in *Arabidopsis thaliana* treated with water, *AtCHS*-dsRNA, or *AtMybL2*-dsRNA (detected at 530 nm). (**a**) Induction of anthocyanin production in water-treated plants (WC) under anthocyanin-induction conditions (+7 °C, 23 h light) in comparison with the control (+22 °C, 16 h light); (**b**) The effect of *AtCHS*-dsRNA treatment on anthocyanin profiles in *A. thaliana*; (**c**) The effect of *AtMybL2*-dsRNA treatment on anthocyanin profiles in *A. thaliana*. A8—Cyanidin 3-*O*-[2′′-*O*-(xylosyl) 6′′-*O*-(*p*-O-(glucosyl) p-coumaroyl) glucoside] 5-*O*-[6′′′-*O*-(malonyl) glucoside]. A10—Cyanidin 3-*O*-[2′′-*O*-(2′′′-*O*-(sinapoyl) xylosyl) 6′′-*O*-(*p*-*O*-(glucosyl) *p*-coumaroyl) glucoside] 5-*O*-glucoside. A11. Cyanidin 3-*O*-[2′′-*O*-(6′′′-*O*-(sinapoyl) xylosyl) 6′′-*O*-(*p*-*O*-(glucosyl)-p-coumaroyl) glucoside] 5-*O*-(6′′′′-*O*-malonyl) glucoside. A7—Cyanidin 3-*O*-[2′′-*O*-(2′′′-*O*-(sinapoyl) xylosyl) 6′′-*O*-(*p*-coumaroyl) glucoside] 5-*O*-glucoside. A5—Cyanidin 3-*O*-[2′′-*O*-(xylosyl)-6′′-*O*-(p-coumaroyl) glucoside] 5-*O*-malonylglucoside. A9—Cyanidin 3-*O*-[2′′-*O*-(2′′′-*O*-(sinapoyl) xylosyl) 6′′-*O*-(*p*-*O*-coumaroyl) glucoside] 5-*O*-[6′′′′-*O*-(malonyl) glucoside]. * — Asterisk indicates a tautomer.

Both HPLC and spectrophotometric analysis of total anthocyanins revealed that plants treated with *AtCHS*-dsRNAs, but not with *NPTII*-dsRNAs, exhibited a markedly lower total content of anthocyanins under the anthocyanin-inducing conditions than the water-treated plants (Figure 3c and Figure S1a). Using HPLC with high-resolution mass spectrometry (HPLC-MS), we detected eight anthocyanin compounds in the water- and dsRNA-treated leaves of *A. thaliana* (Figure 4 and Figure S2a; Table S1). It is possible that other anthocyanins were present in the analyzed tissues of *A. thaliana* but in trace amounts. The content of most individual anthocyanins was higher in the plants grown under the anthocyanin-inducing conditions than at +22 °C (Figure 4a and Figure S2a). Plant treatment with exogenous *AtCHS*-dsRNAs reduced the content of all individual anthocyanins, and the changes were statistically significant for A7, A9, and A11* (Figure 4b and Figure S2a).

### 2.2. AtCHS-Specific Exogenous siRNAs Downregulate AtCHS mRNA Levels and Anthocyanin Content in Arabidopsis

Two 21-nt long complimentary single-stranded RNAs (ss*CHS*-s and ss*CHS*-a) designed to target the *AtCHS* mRNAs were in vitro synthesized and HPLC purified (Table S2). In addition, two 21-nt long complimentary single-stranded RNAs (*NPTII* R3-s-Me and R3-a-Me) designed to target the *NPTII* mRNAs [34] were in vitro synthesized and HPLC purified (Table S2). The complimentary ssRNAs contained a phosphate group at 5′ end, a 2ʹ-*O*-methyl at 3′ end, and 2-nt 3′ overhangs at both ends (Figure 2a,d; Table S2). The ssRNAs were combined and annealed to form siRNAs, i.e., *siCHS* and *siNPTII* (Figure 2a,d). The *NPTII*-specific siRNA was included in the study to verify whether any observed effects of *AtCHS*-siRNA were sequence-specific. In our earlier study, 50 pmol/μL was chosen as the optimal concentration of the *NPTII*-siRNAs for plant foliar treatments due to the combination of effectiveness and lower cost of ssRNA synthesis [34].

**Figure 5:**
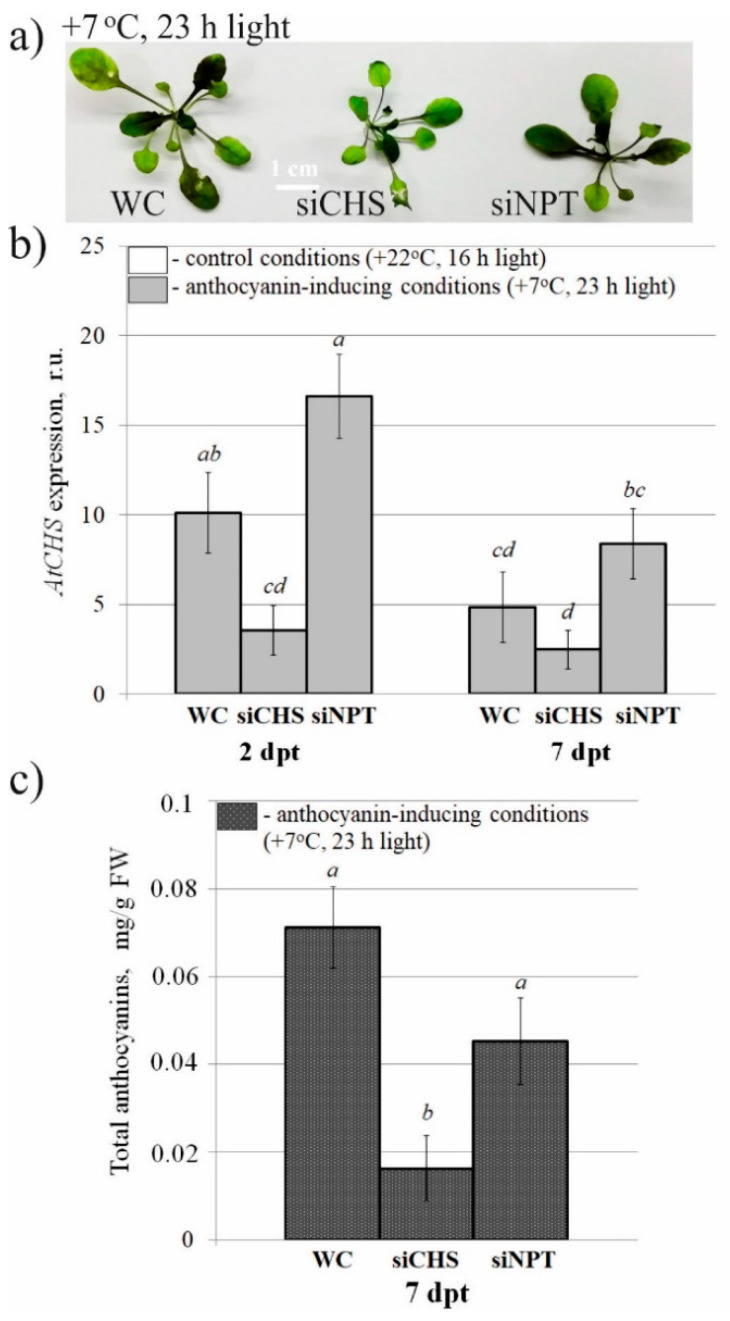
The effect of external *AtCHS*- and *NPTII*-encoding siRNAs on *AtCHS* mRNA level and total anthocyanin content in *Arabidopsis thaliana*. (**a**) *Arabidopsis* plants grown under control (+22 °C, 16 h light, upper panel) and anthocyanin-inducing (+7 °C, 23 h light, lower panel) conditions for seven days after treatment with sterile water or synthetic siRNA. (**b**) Quantitative real-time PCR measuring relative mRNA levels of endogenous *AtCHS* in the leaves of *A. thaliana* treated with water or synthetic siRNAs. (**c**) HPLC results of the total anthocyanin content in the leaves of *A. thaliana* grown at the control and anthocyanin-inducing conditions. WC—*A. thaliana* treated with sterile water; siCHS—*A. thaliana* treated with synthetic *AtCHS*-siRNAs; siNPT—*A. thaliana* treated with *NPTII*-siRNA; dpt—days post-treatment. The data are presented as the mean ± SE (three independent experiments). Means followed by the same letter were not different using Student’s *t* test. *p* < 0.05 was considered statistically significant.

qRT-PCR analysis revealed that the level of *AtCHS* mRNAs was considerably lower in *siCHS*-treated plants than in the water- and *siNPTII*-treated controls grown under the anthocyanin-inducing conditions for two days after the treatments (Figure 5a,b). We noted that the effect became less evident seven days after the treatments. Both HPLC and spectrophotometric analysis of total anthocyanins in the plants cultivated for seven days after the treatments revealed that the si*CHS* treatment led to a markedly lower anthocyanin content under the anthocyanin-inducing conditions than the water and *siNPTII* treatments (Figure 5a,c and Figure S1b). *siNPTII* resulted in the anthocyanin level comparable to that in the water-treated plants. Using HPLC-MS, we also detected eight anthocyanin compounds in the *A. thaliana* treated with water or siRNAs (Figure S2b; Table S1). The si*CHS* treatment considerably lowered content of most individual anthocyanins.

### 2.3. Targeting AtMYBL2 and ANAC032 Repressors by Foliar dsRNA Treatments Downregulates Their mRNA Levels and Leads to a Pronounced AtCHS Upregulation

We used PCR and in vitro transcription protocol to produce dsRNA molecules of the *AtMYBL2* and *AtANAC032* genes, encoding transcriptional repressors negatively regulating anthocyanin biosynthesis in *A. thaliana* [50,51]. Full-length coding cDNAs of the *AtMYBL2* and *AtANAC032* genes were amplified (Figure 2b,c). The obtained PCR products, containing T7 promoters at both ends, were used as templates for in vitro transcription. For external plant treatments, the synthesized dsRNAs were diluted and applied on the leaf surface (on both the adaxial and abaxial sides) of four-week-old wild type *Arabidopsis,* as described above for *AtCHS*-dsRNA. At the same time, we treated *A. thaliana* with *NPTII*-dsRNA to verify whether any observed effects of the dsRNAs were sequence-specific.

**Figure 6:**
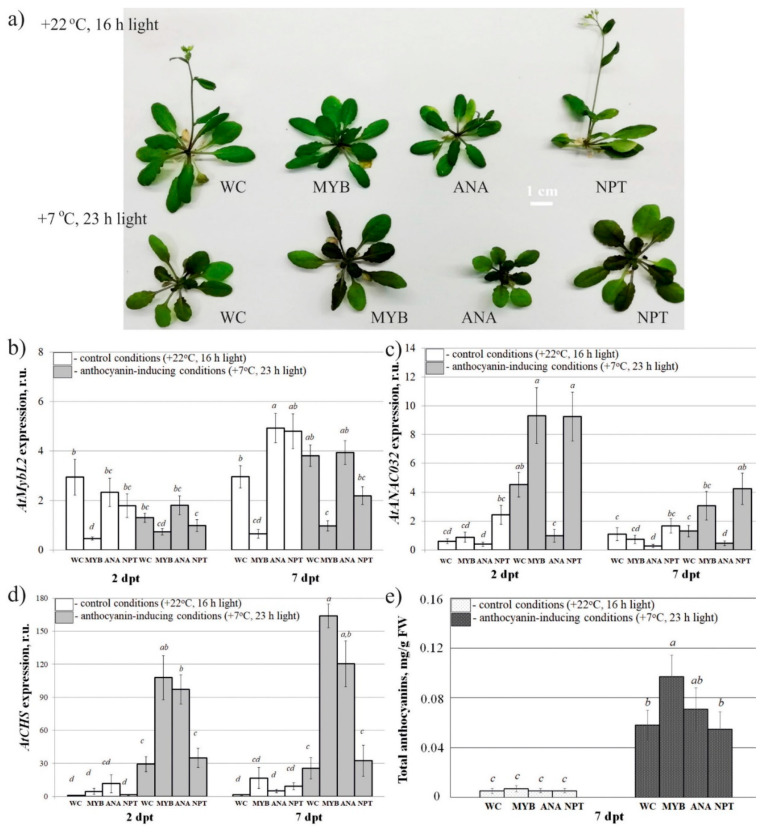
The effect of external *AtMYBL2*-, *AtANAC032-*, and *NPTII*-encoding dsRNAs on *AtMYBL2*, *AtANAC032*, and *AtCHS* mRNA levels and total anthocyanin content in *Arabidopsis thaliana*. (**a**) *Arabidopsis* plants grown under control (+22 °C, 16 h light, upper panel) and anthocyanin-inducing (+7 °C, 23 h light, lower panel) conditions for seven days after treatment with sterile water or synthetic dsRNAs. (**b**) Quantitative real-time PCR measuring relative mRNA levels of endogenous *AtMYBL2* in the leaves of *A. thaliana* treated with water or synthetic dsRNAs. (**c**) Quantitative real-time PCR measuring relative mRNA levels of endogenous *AtANAC032* in the leaves of *A. thaliana* treated with water or synthetic dsRNAs. (**d**) Quantitative real-time PCR measuring relative mRNA levels of endogenous *AtCHS* in the leaves of *A. thaliana* treated with water or synthetic dsRNAs. (**e**) HPLC results of the total anthocyanin content in the leaves of *A. thaliana* grown at the control and anthocyanin-inducing conditions. WC—*A. thaliana* treated with sterile water; MYB—*A. thaliana* treated with *AtMYBL2*-dsRNAs; ANA—*A. thaliana* treated with *AtANAC032*-dsRNAs; NPT—*A. thaliana* treated with *NPTII*-dsRNA; dpt—days post-treatment. The data are presented as the mean ± SE (three independent experiments). Means followed by the same letter were not different using Student’s *t* test. *p* < 0.05 was considered statistically significant.

Then, we studied whether simple exogenous application of the *AtMYBL2*-, *AtANAC032*-, and *NPTII*-dsRNAs to the foliar surface of four-week-old *A. thaliana* could lead to any changes in the mRNA transcript levels of *AtMYBL2*, *AtANAC032*, and *AtCHS* genes in comparison with the water-treated controls two and seven days post-treatments (Figure 6a). For this purpose, we also divided the treated *A. thaliana* rosettes into two groups for incubation under control conditions (+22 °C, 16 h light) and anthocyanin-inducing conditions (+7 °C, 23 h light). Foliar plant treatment with the *AtMYBL2*-dsRNAs triggered considerable downregulation of *AtMYBL2* mRNA levels both under standard and anthocyanin-inducing conditions (Figure 6b). *AtMYBL2* transcript levels were significantly reduced two and seven days post-treatment at +22 °C and seven days post-treatment at +7 °C. Exogenous application of *AtANAC032*-dsRNA also resulted in a pronounced inhibition of *AtANAC032* mRNA levels, especially under the anthocyanin-inducing conditions (Figure 6c). Further analysis revealed a dramatic upregulation of *AtCHS* expression after application of both *AtMYBL2*- and *AtANAC032*-dsRNAs (Figure 6d). However, the dsRNA-induced effect on anthocyanin accumulation was not as evident as that for gene transcript levels (Figure 6e and Figure S1c). While the *AtMYBL2*- and *AtANAC032*-dsRNA treatments led to a 3.3-6.4-fold upregulation of *AtCHSs,* the total content of anthocyanins was considerably increased only after foliar treatment with *AtMYBL2* and by 1.7-fold (Figure 6d,e). HPLC analysis of individual anthocyanins revealed a statistically considerable increase in the content of A8, A11, and A9 after treatment with *AtMYBL2*-dsRNA (Figure S2c). Importantly, exogenous application of *NPTII*-dsRNA did not have a marked effect on the *AtMYBL2*, *AtANAC032*, or *AtCHS* mRNA levels.

### 2.4. Detection of AtCHS-dsRNA in A. thaliana Leaves by Laser Scanning Microscopy

**Figure 7:**
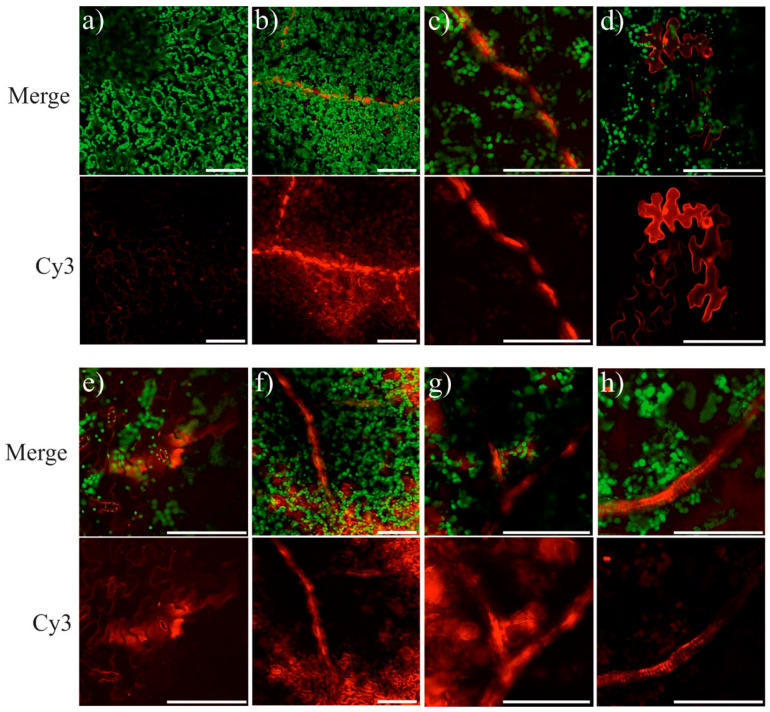
Detection of Cy3-labeled *AtCHS-dsRNA* (red) in the externally treated *Arabidopsis thaliana* leaves by laser scanning microscopy. Adaxial (**a**–**c**) and abaxial (**d**–**h**) leaf sides of four-week old *A. thaliana* were exogenously treated with the labeled *AtCHS*-dsRNA (red) by sterile soft brushes and analyzed 13–15 h post-treatment. (**a**) Negative control (sterile water); (**b**–**h**)—*AtCHS*-dsRNA treatment. Green color results from the autofluorescence of chloroplasts. Red color results from the Cy3-labeled dsRNA. Scale bars, 100 μm. Three independent trials showed the same results.

To further investigate localization and transport of the exogenous dsRNAs, we labeled the in vitro synthesized *AtCHS-*dsRNAs with the Cy3 dye and applied to the adaxial and abaxial foliar surface of four-week-old *A. thaliana* rosettes at 21:00 at a concentration of 0.35 µg/µL [31] by spreading with sterile individual soft brushes. Linear unmixing of the λ-stacks revealed two peaks of fluorescence, including a peak at ≈570 nm–580 nm and a peak at ≈690 nm (Figure S3). The peak at 570 nm–580 nm corresponds to Cy3 (Silencer™ siRNA Labeling Kit with Cy™3 dye), and the peak at 690 nm—chlorophyll fluorescence [52]. Inspection of the adaxial and abaxial leaf surface by laser scanning microscopy 13–15 h post-treatment detected the presence of the Cy3-labeled dsRNA in the leaf veins, parenchyma cells, and stomata of the dsRNA-treated *A. thaliana* leaves (Figure 7).

## 3. Discussion

It is well established that spraying plants with dsRNAs and siRNAs encoding key genes of plant pathogenic fungi [16,17,18,19,20,21] and viruses [22,23,24,25,26,27] effectively reduces development of the pathogens and suppresses the infection process. These externally applied dsRNAs and siRNAs have been shown to spread systemically into plant tissues and were uptaken by the fungal cells inducing a RNAi-mediated silencing of the targeted genes of the pathogens [16,17,21,27]. This strategy of plant disease control was termed as spray-induced gene silencing (SIGS) and is currently considered as an efficient and sustainable plant protection strategy and for other crop improvement strategies [29]. Much less is known about the influence of exogenous dsRNAs/siRNAs on gene silencing in the plant genome. Only four studies [41,42,43,44] and one patent [40] reported on direct plant treatments with naked dsRNA resulting in a reduction of the mRNA levels of a plant endogenous gene target that would also lead to phenotypic or biochemical changes. In addition, two studies reported using nanoparticles [45] or laser light [46] to ensure perception of exogenous dsRNA and downregulation of a plant endogenous gene by external dsRNA application.

We developed our study based on the initial report by Numata et al. [32] who infiltrated *A. thaliana* leaves with a carrier peptide in a complex with siRNAs encoding the *AtCHS* gene. Numata et al. [32] reported a local loss of anthocyanin pigmentation by visual observation, but *AtCHS* mRNA and anthocyanin levels were not analyzed. In our study, we show that foliar application of the gene-specific dsRNAs and siRNAs highly reduced mRNA levels of three anthocyanin biosynthesis-related genes and considerably affected anthocyanin production at both two days and seven days post-treatment. The present study demonstrated that external dsRNA treatments could lead to both gene downregulation (exogenous *AtCHS*-dsRNAs downregulated *AtCHS* expression) and gene upregulation (*AtMYBL2-dsRNAs* and *AtANAC032*-dsRNAs upregulated *AtCHS* expression). In all experiments, we treated *A. thaliana* with *NPTII*-encoding dsRNAs, which encode a bacterial gene that is not encoded in the genome of wild-type *Arabidopsis*. In all cases, the foliar-applied unspecific *NPTII*-dsRNAs had no effect on the expression of the *AtCHS*, *AtMYBL2*, and *AtANAC032* genes of *A. thaliana*, which proves that the observed dsRNA-induced gene silencing effect was sequence-specific and was not a result of the dsRNA application itself.

The positive transcriptional regulation of anthocyanin biosynthesis in *Arabidopsis* and other plants is achieved via a concerted action of a number of transcription factors, involving the MYB–bHLH–WD repeat (MBW) protein complex, which is composed of R2R3-MYB, basic helix-loop-helix (bHLH), and WD40-repeat proteins [53]. As to negative regulation, single repeat R3-MYB transcription factors, including MYBL2, CAPRICE (CPC), TRIPTYCHON (TRY), ENHANCER OF TRY AND CPC 1 (ETC1), and ETC2, were shown to suppress anthocyanin accumulation by interfering with the formation of MBW protein complex [50,53]. In addition, there are other molecular players regulating anthocyanin biosynthesis in *Arabidopsis*, such as ubiquitin protein ligases or other transcription factors. For example, a NAC transcription factor, ANAC032, that has recently been reported to act as a negative regulator of anthocyanin biosynthesis in *Arabidopsis thaliana* during stress conditions. In our work, we targeted two unrelated transcription repressors, MYBL2 and ANAC032, and verified that the gene-specific dsRNAs downregulated their expression and upregulated *AtCHS* at the same time. According to our data, both the *AtMYBL2*- and *AtANAC032-dsRNA* treatments led to a 3.3-6.4-fold upregulation of *AtCHS* expression, while the total content of anthocyanins was considerably increased only after foliar treatment with *AtMYBL2* and by 1.7-fold. We propose that since there is a plethora of transcriptional regulators implicated in the regulation of anthocyanin biosynthesis, the resulting elevation of anthocyanin production after targeting only two of them was not dramatic.

In summary, our results demonstrate a high potential of exogenous dsRNAs for regulating plant endogenous genes due to a pronounced sequence-specific gene downregulation effect. Furthermore, external plant treatments with gene-specific dsRNAs were capable of inducing the desired phenotypic and biochemical effects. Taken together, the findings reveal that exogenous RNAs can be exploited by plant biologists for further crop improvement and for fundamental gene functional studies.

## 4. Materials and Methods

### 4.1. Plant Material and Growth Conditions

The seeds of wild-type *A. thaliana* (cv. Columbia) were vapor-phase sterilized as described [34] and plated on solid ½ Murashige and Skoog (MS) medium for two days at 4 °C. Then, the plates were kept at 22 °C for 1 week in a growth chamber (Sanyo MLR-352, Panasonic, Osaka, Japan) at a light intensity of ~120 μmol m^−2^ s^−1^ over a 16 h daily light period. One-week-old *A. thaliana* seedlings were planted to pots (7 cm × 7 cm) containing 100 g of commercially available rich soil (the soil was well-irrigated by filtered water applied at the bottom of the pots). Then, the plants were grown in the chamber at 22 °C under plastic wrap for additional three weeks without additional irrigation before RNA treatments of four-week-old plants. After the RNA treatments, the *A. thaliana* was incubated for additional seven days either under control (+22 °C, 16 h daily light period) or anthocyanin-inducing (+7 °C, 23 h daily light period) conditions in a growth chamber (KS-200, Smolenskoye SKTB SPU, Smolensk, Russia) without further irrigation to induce *AtCHS* expression and anthocyanin accumulation.

### 4.2. Isolation and Sequencing of AtCHS, AtMybL2, and AtANACO323 Transcripts

Full-length coding cDNA sequences of *AtCHS* (AT5G13930.1, 1188 bp) *AtMYBL2* (AT1G71030.1, 588 bp), and *AtANAC032* (AT1G77450, 762 bp) were amplified by RT-PCR using RNA samples extracted from the adult leaves of *A. thaliana*. The RT-PCRs were performed in a Bis-M1105 Thermal Cycler (Bis-N, Novosibirsk, Russia). The primers are listed in Table S3. The RT-PCR products were subcloned into pJET1.2/blunt and sequenced as described previously [54].

### 4.3. dsRNA Synthesis and Application

All dsRNAs were synthesized using the T7 RiboMAX™ Express RNAi System (Promega, Madison, WI, USA). For this purpose, the cloned full-length cDNAs of *AtMYBL2* and AtANAC032 and a large cDNA fragment of *AtCHS* (736 bp out of 1188 bp) were amplified by PCR for in vitro transcription and dsRNA production. We also amplified a large fragment of *NPTII* (GenBank AJ414108, 599 bp out of 798 bp) using pZP-RCS2-nptII plasmid [55]. The T7 promoter sequence was introduced into both the 5′ and 3′ ends of the amplified *AtCHS*, *AtMYBL2*, *AtANAC032*, or *NPTII* in a single PCR for each gene using primers listed in Table S3. The PCRs were performed in the Bis-M1105 Thermal Cycler programmed according to T7 RiboMAX™ Express RNAi System instructions. Then, the obtained PCR products were used as templates for in vitro transcription and dsRNA synthesis following the manufacturer’s protocol. The resultant dsRNAs were analyzed by gel electrophoresis and spectrophotometry to estimate dsRNA purity, integrity, and amount.

The *AtCHS-*, *AtMYBL2-*, *AtANAC032*-, and *NPTII*-dsRNAs were applied to individual four-week-old rosettes of wild-type *A. thaliana* by spreading with individual soft brushes (natural pony hair) sterilized by autoclaving [31] (Supplementary Video S1). For each dsRNA treatment, 35 µg of the dsRNA were diluted in 100 µL of nuclease-free water and applied to the foliar surface (all leaves of one rosette for each type of condition were treated on both the adaxial (upper) and abaxial (lower) sides). One plant of *A. thaliana* was treated with the dsRNA of each type (100 µL) and one plant—with sterile filtered water (100 µL) in each independent experiment. The dsRNAs in all experiments were applied to four-week-old rosettes of *A. thaliana* at a late day time (21:00–21:30) under low soil moisture conditions, since the appropriate plant age, late day time, and low soil moisture at the time of dsRNA application were important parameters for successful *NPTII* suppression in transgenic *A. thaliana* according to our recent analysis [31]. Soil water content before dsRNA treatment was 50–60%.

### 4.4. siRNA Synthesis and Application

The *AtCHS*- and *NPTII*-encoding ssRNAs were *in vitro*-synthesized, modified, and HPLC purified by Syntol (Moscow, Russia). The RNA oligonucleotide sequences are presented in Table S2 and Figure 2a,d. The synthesized single-stranded oligonucleotides were prepared to form siRNA as described [34]. Briefly, to form the siRNA duplexes, equal volumes of the ssRNAs diluted to a concentration of 100 pmol/µL were combined and annealed at 90 °C for 1 min. Then, the mixture was slowly cooled to room temperature. The final concentration of annealed oligonucleotides was 50 pmol/µL. 100 µL of each siRNA duplex or 100 µL of nuclease-free water were applied onto the leaf surface of four-week-old *A. thaliana* by spreading with individual soft brushes as described above for dsRNA.

### 4.5. RNA Isolation and Reverse Transcription

For RNA isolations, a typical adult leaf of *A. thaliana* was collected from the same individual plant before treatment, two days and seven days post-treatment for each type of treatment in an independent experiment. Total RNA was isolated using the cetyltrimethylammonium bromide (CTAB)-based protocol [56] and complementary DNAs were synthesized as described [57].

### 4.6. Gene Expression Analysis by qRT-PCR

The reverse transcription products were amplified by PCR and verified for the absence of DNA contamination, using primers listed in Table S3. The qRT-PCRs were performed with SYBR Green I Real-time PCR dye and a real-time PCR kit (Evrogen, Moscow, Russia) as described [58] using two internal controls (GAPDH and UBQ) selected in previous studies as relevant reference genes for qRT-PCRs on *Arabidopsis* [59]. The expression was calculated by the 2−ΔΔCT method [60]. All gene identification numbers and used primers are listed in Table S3.

### 4.7. Quantification of Anthocyanins

Anthocyanin content in *A. thaliana* rosettes was determined using a SPECTROstar Nano spectrophotometer (BMG Labtech, Ortenberg, Germany) as described in Teng et al. [61]. Treated *A. thaliana* rosettes were frozen at −20 °C and subsequently homogenized using a mortar and a pestle. Shredded tissue was weighed and extracted for 1 d at 4 °C in 1 mL of 1% (*v/v*) hydrochloric acid in methanol. Then, the mixture was centrifuged at 13,200 rpm for 15 min and the absorbance of the supernatant was measured at 530 and 657 nm. Relative anthocyanin concentrations were calculated as described [61].

For HPLC–MS analysis, the samples were filtered through a 0.45-um nylon filter. Identification of all anthocyanins was performed using a 1260 Infinity analytical HPLC system (Agilent Technologies, Santa Clara, CA, USA) coupled to Bruker HCT ultra PTM Discovery System (Bruker Daltonik GmbH, Bremen, Germany) equipped with an electrospray ionization (ESI) source. The data for anthocyanins were acquired in positive ion mode under the operating conditions as described [62]. The MS spectra were recorded across an *m/z* range of 100–1500, and the individual anthocyanins were identified as described [63].

HPLC with diode array detection (HPLC–DAD) for quantification of all anthocyanins was performed using a HPLC LC-20AD XR analytical system (Shimadzu, Kyoto, Japan). DAD data were recorded in the 200–800 nm range, and chromatograms for quantification were acquired at 530 nm. The chromatographic separation was performed on Shim-pack GIST C18 column (150 mm, 2.1-nm i.d., 3-µm part size; Shimadzu, Japan). Anthocyanins were separated using 0.1% formic acid and acetonitrile as mobile phases A and B, respectively, with the following elution profile: 0 to 35 min 0% of B; 35 to 40 min 40% of B; 40 to 50 min 50% of B; 50 to 65 min 100% of B. 5 μL of the sample extract was injected with a constant column temperature maintained at 40 °C and a flow rate maintained at 0.2 mL/min. All solvents were of HPLC grade. The contents of anthocyanins were determined by external standard method using the four-point regression calibration curves built with the available standards. The commercial standard cyanidin chloride was obtained from Sigma-Aldrich (St. Louis, MO, USA) and used as the control.

### 4.8. Fluorescent dsRNA Labeling and Laser Scanning Microscopy

Fluorescent labeling of the in vitro synthesized *AtCHS*-dsRNA was performed using the Silencer™ siRNA Labeling Kit with Cy™3 dye (Thermo Fisher Scientific, Waltham, MA, USA) following the manufacturer’s instructions. A total of 35 µg of the labeled dsRNA (100 µL) was applied on the adaxial and abaxial leaf surface of four-week *A. thaliana* as described above. Fluorescent signals were analyzed 13–15 h after the foliar plant treatments by laser scanning microscopy. The whole leaves were mounted in distilled water in a Petri dish and were observed under a Zeiss LSM 780 laser scanning microscope operated in λ-mode equipped with a Plan-Apochromat 20×/0.8 and a Plan-Neofluar 40×/0.6 objectives. The excitation wavelength of an argon laser was set at 488 nm and the emission signal was registered at 20 evenly spaced wavelengths (8.9 nm apart) in a range from 500 to 693 nm by using a QUASAR detector. Finally, the resulted λ-stacks were linearly unmixed with the Zeiss Zen 2.1 SP3 (Black Edition) software. The laser scanning microscopy was carried out at the Far Eastern Center of Electron Microscopy (A.V. Zhirmunsky National Scientific Center of Marine Biology, FEB RAS, Vladivostok, Russia).

### 4.9. Statistical Analysis

The data are presented as mean ± standard error (SE) and were tested by paired Student’s *t*-test. The *p* < 0.05 level was selected as the point of minimal statistical significance in all analyses. At least three independent experiments were performed for each type of analysis.

## Data Availability

The data presented in this study are available within the article and Supplementary Materials.

## Supplementary Materials

Supplementary Materials can be found at https://www.mdpi.com/article/10.3390/ijms22136749/s1.
