# Supplementary material for: External dsRNA Downregulates Anthocyanin Biosynthesis-Related Genes and Affects Anthocyanin Accumulation in Arabidopsis thaliana
Source: Int J Mol Sci. 2021 Jun 23;22(13):6749. doi: 10.3390/ijms22136749 (PMC8269191; doi:10.3390/ijms22136749)
Supplement: Supplementary file 1 [file ijms-22-06749-s001.zip › Supplementary.pdf]

Supplementary Table S1 List of anthocyanins identified in the methanol extracts of *A. thaliana* rosettes.

Peak	Rt (min)[a]	UV, λmax (nm)	MS (m/z)	MS^2 fragments (m/z)	Assignment
1	19,7	527	1137	535,889	A8
2	20,8	520	1258	1095,449	A10
3	21,8	540	1344	1095,535	A11
4	22,8	535	1344*	1095,535	A11*
5	24,1	530	1095	933,449	A7
6	25	525	975	727,535,287	A5
7	26	530	1182	1137,933,535,491	A9
8	26,8	535	1182*	1137,933,535,491	A9*

*Asterisk indicates a tautomer.

A8 – Cyanidin 3-O-[2″-O-(xylosyl) 6″-O-(p-O-(glucosyl) p-coumaroyl) glucoside] 5-O-[6‴-O-(malonyl) glucoside].
A10 – Cyanidin 3-O-[2″-O-(2‴-O-(sinapoyl) xylosyl) 6″-O-(p-O-(glucosyl) p-coumaroyl) glucoside] 5-O-glucoside.
A11. Cyanidin 3-O-[2″-O-(6‴-O-(sinapoyl) xylosyl) 6″-O-(p-O-(glucosyl)-p-coumaroyl) glucoside] 5-O-(6⁗-O-malonyl) glucoside.
A7 – Cyanidin 3-O-[2″-O-(2‴-O-(sinapoyl) xylosyl) 6″-O-(p-coumaroyl) glucoside] 5-O-glucoside.
A5 – Cyanidin 3-O-[2″-O-(xylosyl)-6″-O-(p-coumaroyl) glucoside] 5-O-malonylglucoside.
A9 – Cyanidin 3-O-[2″-O-(2‴-O-(sinapoyl) xylosyl) 6″-O-(p-O-coumaroyl) glucoside] 5-O-[6⁗-O-(malonyl) glucoside].

Supplementary Table S2 *In vitro*-synthesized single-stranded RNA olignucleotides.

	RNA oligonucleotide name	5' - 3' RNA oligonucleotides	Modification
1	ssCHS-s	5′-GUGACUGGAACUCCCUCUUCU	5′ phosphate
2	ssCHS-a	5′-AAGAGGGAGUUCCAGUCACUU	2′-*O*-methyl at 3′ end
3	NPTII (R3-s-Me)	5'-AAUGGCCGCUUUUCUGGAUUC	5′ phosphate
4	NPTII (R3-a-Me)	5'-AUCCAGAAAAGCGGCCAUUUU	2′-*O*-methyl at 3′ end

Supplementary Table S3 Primers used in RT-PCR and qRT-PCRs.

Gene name (ID number)	Primer name	Primers, 5′-3′
Primers for cloning full-length cDNA coding sequences, 5′-3′		
AtCHS (AT5G13930.1)	AtCHS-Nach-S<br>AtCHS-konA	5′ATGGTGATGGCTGGTGCTTCTT<br>5′TTAGAGAGGAACGCTGTGCAAG
AtMybL2 (AT1G71030.1)	AtMybL2-s1<br>AtMybL2-kon	5′ATGAACAAAACCCGCCTTC<br>5′TCATCGGAATAGAAGAAGC
AtANAC032 (AT1G77450.1)	ANAC032-s1<br>ANAC032-kon	5′ATGATGAAATCTGGGGCTG<br>5′TCAGAAAGTTCCCTGCCTA
Specific primers for dsRNA design, 5′-3′		
AtCHS (AT5G13930.1)	AtCHS-RNAs2<br>AtCHS- RNA-a2	5'TAATACGACTCACTATAGGGAGAGCTTCTTGGTCTCC GTCCTTCC<br>5'TAATACGACTCACTATAGGGAGATTAGAGAGGAACG CTGTGCAAG
AtMybL2 (AT1G71030.1)	MybL2-dsRNA-s1<br>MybL2-dsRNA-a1	5'TAATACGACTCACTATAGGGAGAATGAACAAAACCC GCCTTC<br>5'TAATACGACTCACTATAGGGAGATCATCGGAATAGA AGAAGCGTTT

AtANAC032 (AT1G77450.1)	ANA-dsRNA-s1<br><br>ANA-dsRNA-a1	5'TAATACGACTCACTATAGGGAGAATGATGAAATCTGGGGCTGATTTG<br><br>5'TAATACGACTCACTATAGGGAGATCAGAAAGTTCCCTGCCTAACC
NPTII (AY818371)	npt-T71-s<br><br>npt-T72-a	5'TAATACGACTCACTATAGGGAGAATGTGGATTGAACAAGATGGATTG<br><br>5'TAATACGACTCACTATAGGGAGATCCACCATGATATTCGGCAAGCAG
Primers for cDNA check-up on DNA contamination, 5′-3′		
AtGAPDH (GenBank NM_111283)	AtGapdh-s<br>AtGapdh-a	5′CTG GAA TGT CTT TCC GTG TC<br>5′ATT CGT TGT CGT ACC ATG AC
Primers for PCR and real-time PCR, 5′-3′		
AtCHS (AT5G13930.1)	AtCHS-Nach-S<br>AtCHS-realA	5′ATGGTGATGGCTGGTGCTTCTT<br>5′- CACATGGTTCTCAGGGTTAGC
AtMybL2 (AT1G71030.1)	AtMybL2-reals1<br>MybL2-3UTR-realA1	5′TTGCCTGACCTAAACATTG<br>5′GCCGGTCCAATTCAGGATTAAC
AtANAC032 (AT1G77450.1)	ANAC032-reals1<br>AtANA-3UTR-real-a1	5′CGTTTAATTACGTAGATGC<br>5′CACTTTCCACTAACTCTAATCGC
NPTII (GenBank AJ414108)	nptII-realS<br><br>nptII-realA	5′TTGCTGAAGAGCTTGGCGGCGAAT<br><br>5′TCAGAAGAACTCGTCAAGAAGG
AtGAPDH (GenBank NM_111283)	AtGapdh-real-s<br>AtGapdh-real-a	5′TTG GTG ACA ACA GGT CAA GCA<br>5′AAA CTT GTC GCT CAA TGC AAT
AtUBQ (GenBank NM_001084884)	AtUBQ-realS<br>AtUBQ-realA	5′GGCCTTGTATAATCCCTGATGAATAAG<br>5′AAAGAGATAACAGGAACGGAAACATAGT

Supplementary Fig. S1 Spectrophotometric results of total anthocyanins in the rosettes of *A. thaliana* grown under the control (+22°C, 16 h light) and anthocyanin-inducing (+7°C, 23 h light) conditions for seven days. (a) total anthocyanins in *A. thaliana* treated with sterile water or synthetic *AtCHS*- or *NPTII*-dsRNAs; (b) total anthocyanins in *A. thaliana* treated with sterile water or synthetic si*CHS* or si*NPTII*; (c) total anthocyanins in *A. thaliana* treated with sterile water or synthetic *MYBL2*- or *ANAC032*-dsRNAs.
WC – *A. thaliana* treated with sterile water, CHS – *A. thaliana* treated with *AtCHS*-dsRNAs, NPT – *A. thaliana* treated with *NPTII*-dsRNAs; siCHS - *A. thaliana* treated with *AtCHS*-siRNAs; siNPTII - *A. thaliana* treated with *NPTII*-siRNAs; MYB – *A. thaliana* treated with *AtMYB*-dsRNAs; ANA – *A. thaliana* treated with *AtANAC032*-dsRNAs; dpt – days post treatment.
One anthocyanin unit equals one absorbance unit [A530 - (1/4A657)] in 1 mL of extraction solution.
The data are presented as the mean ± SE (four independent experiments). Means followed by the same letter were not different using Student's *t* test. $P \leq 0.05$ was considered statistically significant.

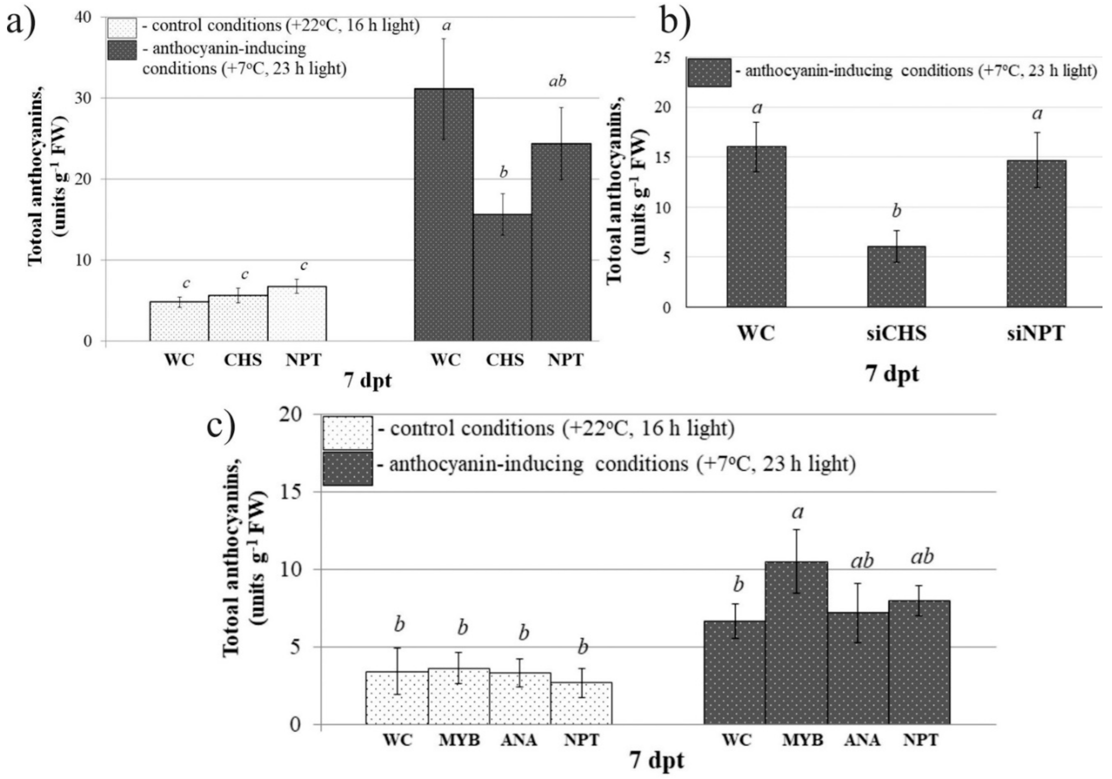

Supplementary Fig. S2 HPLC profile of anthocyanins in the leaves of *Arabidopsis thaliana* grown under the control (+22°C, 16 h light) and anthocyanin-inducing (+7°C, 23 h light) conditions. WC – *A. thaliana* treated with sterile water, dsCHS – *A. thaliana* treated with *AtCHS*-dsRNAs, dsNPT – *A. thaliana* treated with *NPTII*-dsRNA. The data are presented as the mean ± SE (three independent experiments). Means followed by the same letter were not different using Student's t test. $p < 0.05$ was considered statistically significant. * — Asterisk indicates a tautomer.

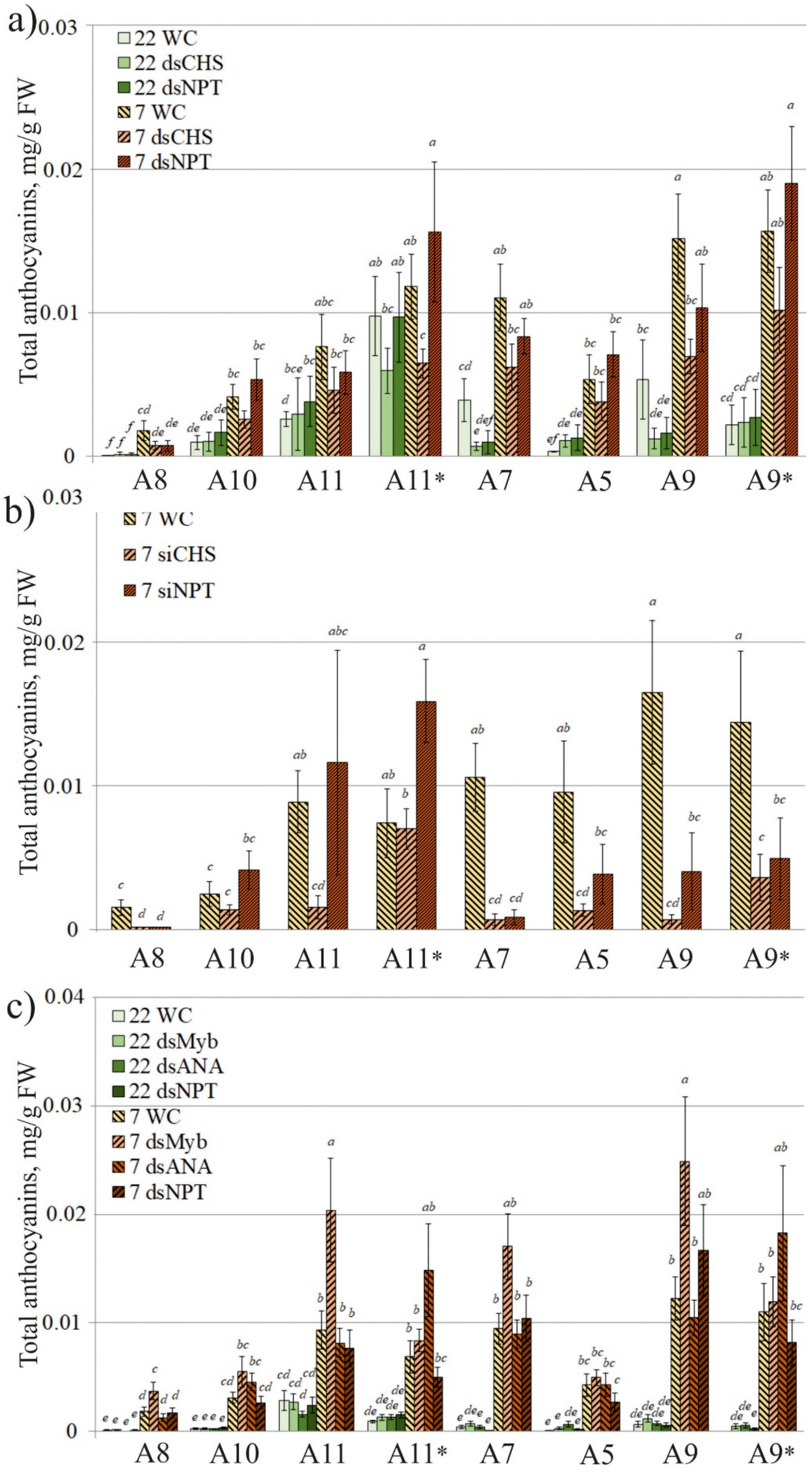

Supplementary Fig. S3 Emission spectrum of Cy3 and chlorophyll obtained in the λ-scanning mode of Zeiss LSM 780 laser scanning microscope. Cy3 fluorescence (peak at ≈ 570 nm–580 nm) is highlighted in green and chlorophyll fluorescence (peak at ≈ 690 nm) is highlighted in red.

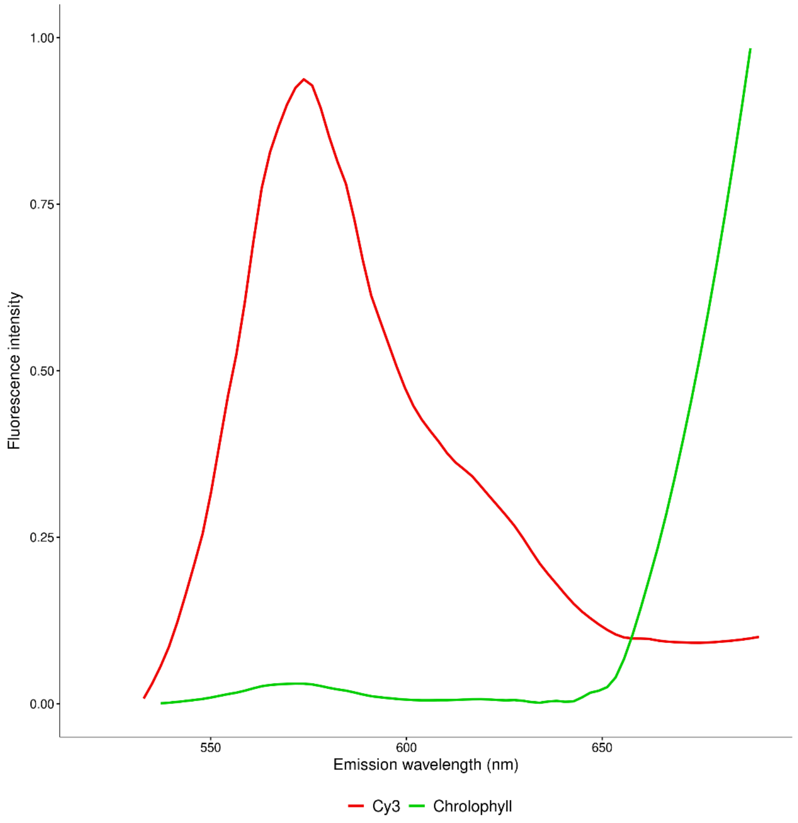

Supplementary Video S1

The dsRNAs were applied on the foliar surface of four-week-old *Arabidopsis thaliana* by spreading with individual soft brushes (natural pony hair) sterilized by autoclaving. All leaves of one rosette for each type of condition were treated on both the adaxial (upper) and abaxial (lower) sides. The leaf surface of each individual plant was treated with 0.35 μg/μL of dsRNA or water (100 μL per individual plant). For each dsRNA treatment, 35 μg of the dsRNA were diluted in 100 μL of nuclease-free water.
