# Supplementary figures and images for: External dsRNA Downregulates Anthocyanin Biosynthesis-Related Genes and Affects Anthocyanin Accumulation in Arabidopsis thaliana
Source: Int J Mol Sci. 2021 Jun 23;22(13):6749. doi: 10.3390/ijms22136749 (PMC8269191; doi:10.3390/ijms22136749)

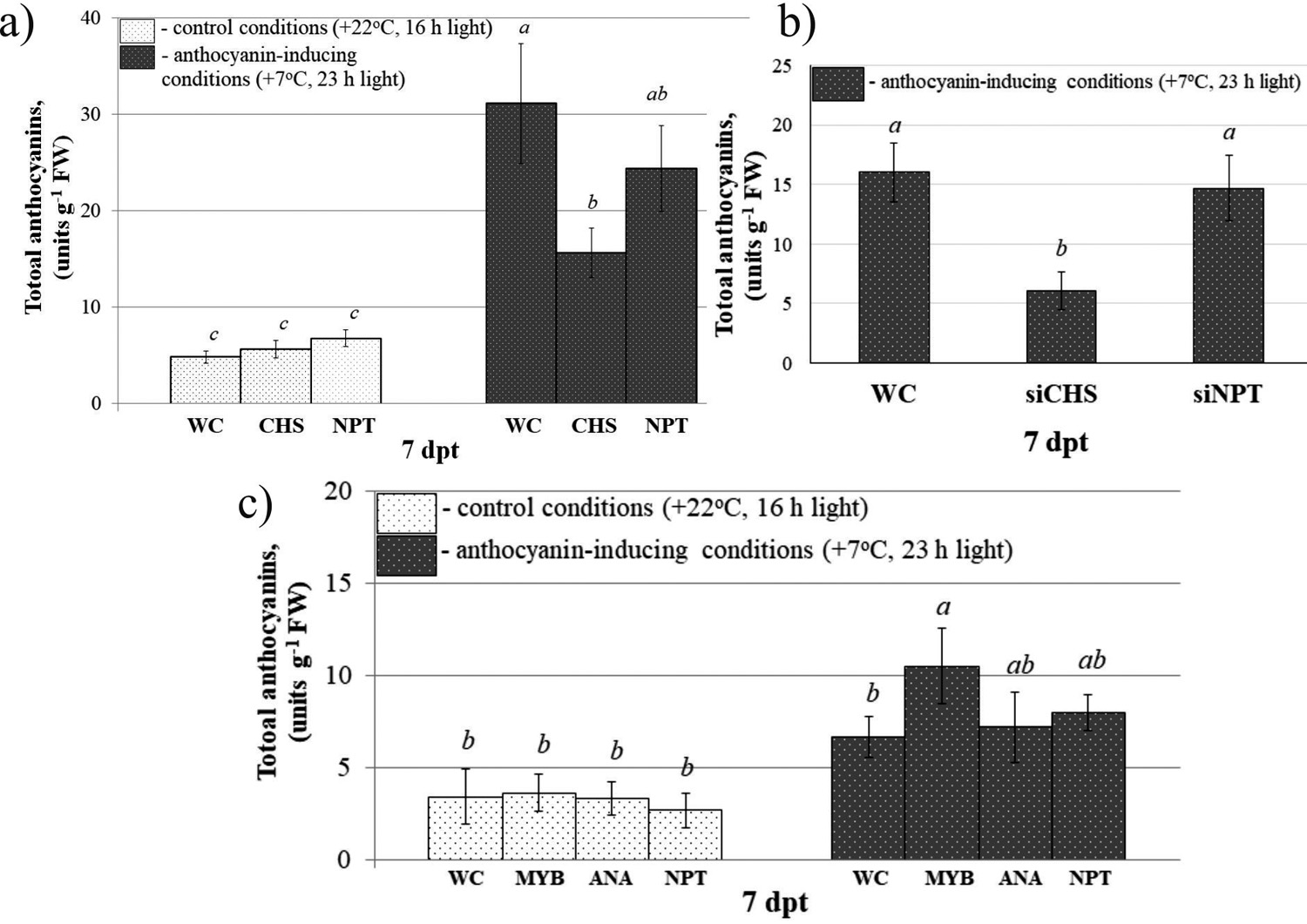

Supplement: Supplementary file 1 [file ijms-22-06749-s001.zip › Figure S1.jpg]

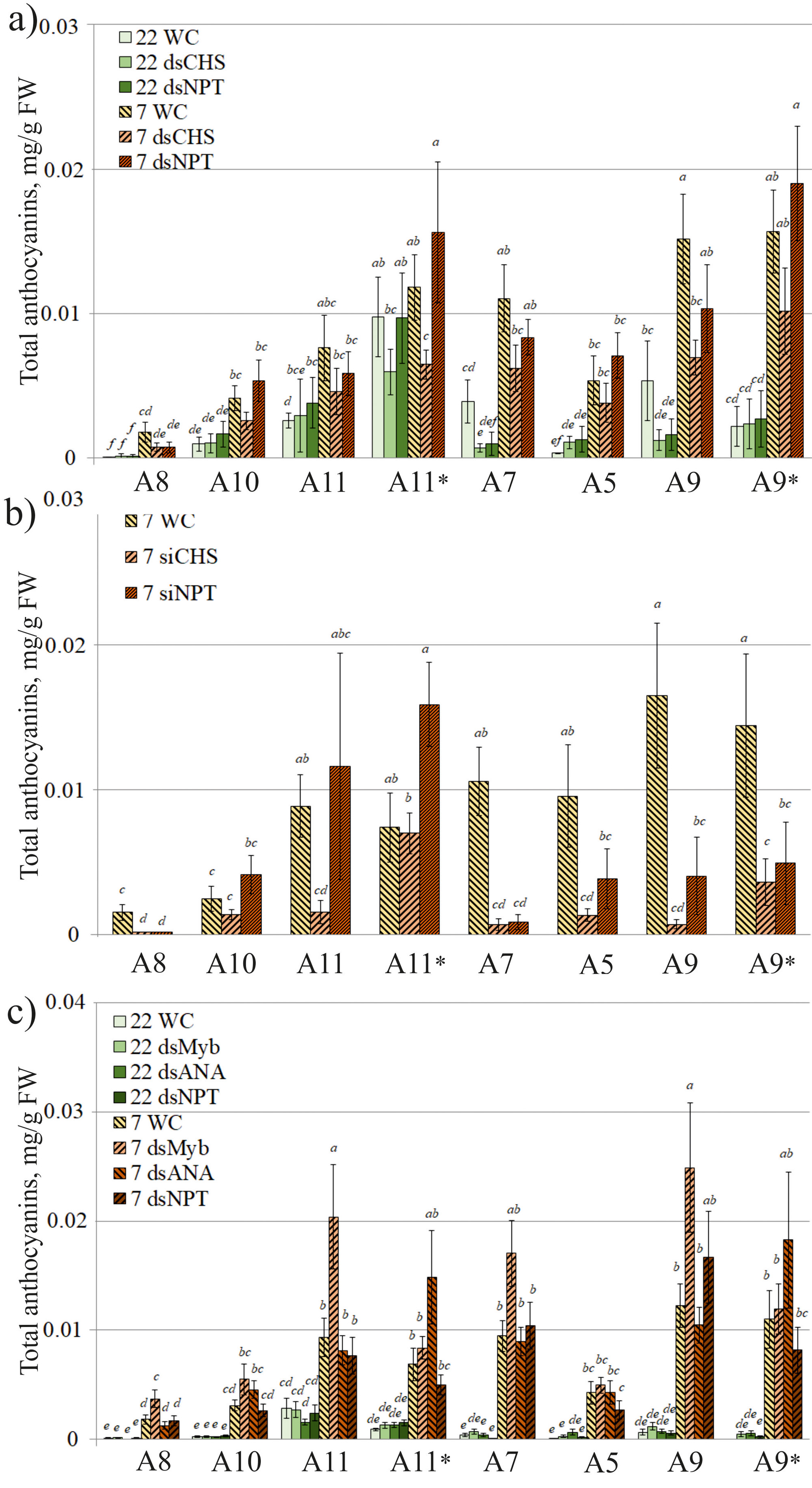

Supplement: Supplementary file 1 [file ijms-22-06749-s001.zip › Figure S2.jpg]

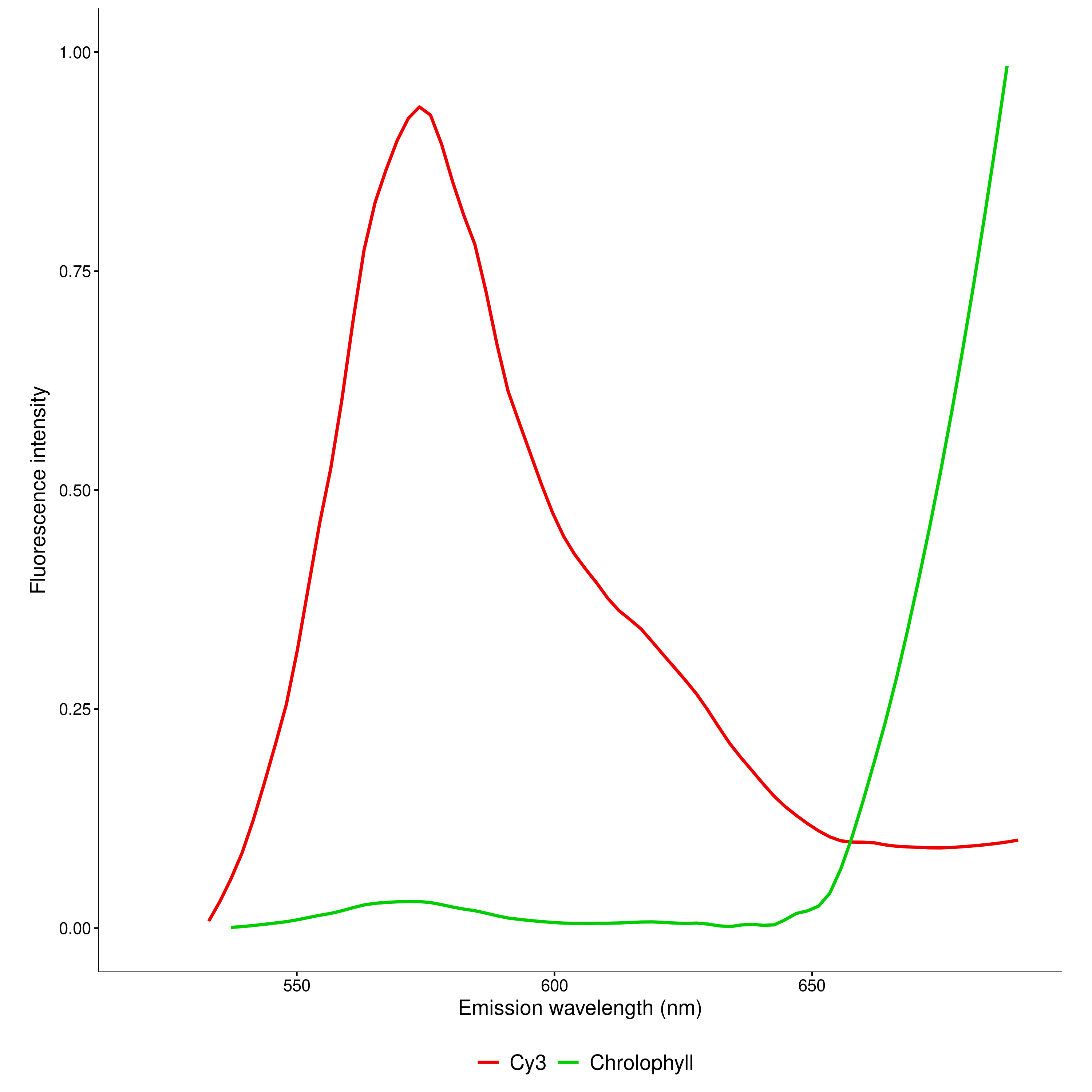

Supplement: Supplementary file 1 [file ijms-22-06749-s001.zip › Figure S3.tiff]
